# A Review of Bioinformatics Tools to Understand Acetaminophen-Alcohol Interaction

**DOI:** 10.3390/medicines6030079

**Published:** 2019-07-25

**Authors:** Bryan Hedgpeth, Roy Missall, Anna Bambaci, Matthew Smolen, Sevgi Yavuz, Jessica Cottrell, Tinchun Chu, Sulie L. Chang

**Affiliations:** 1 Department of Biological Science, Seton Hall University, South Orange, NJ 07079, USA; 2 The Institute of NeuroImmune Pharmacology (I-NIP), Seton Hall University, South Orange, NJ 07079, USA

**Keywords:** alcohol, drug-ethanol interaction, acetaminophen, bioinformatics, pathways

## Abstract

**Background:** Drug-ethanol interaction can result in hepatotoxicity. The liver is capable of metabolizing both acetaminophen and ethanol; however, severe acute or moderate chronic simultaneous exposure can cause cell and tissue damage. Therapeutic doses can become harmful if gene activity is altered via competition for metabolic pathways. Simultaneous intake of ethanol and acetaminophen results in overactive CYP2E1 and depletion of glutathione, leaving NAPQI to build up in the liver. NAPQI is a hepatotoxic substance typically neutralized by glutathione. **Methods:** Bioinformatics tools including PharmGKB, Chemical Annotation Retrieval Toolkit, Transcriptome Analysis Console 4.0 (TAC), wikipathways, STRING, and Ingenuity Pathway Analysis (IPA) were used to explore interactive metabolic pathways of ethanol-acetaminophen exposure as a proof of concept for assessing drug-drug or drug-alcohol interactions. **Results:** As the ethanol-acetaminophen comparison indicates, bioinformatics tools may be used to understand interactive pathways following exposure to ethanol and acetaminophen, with potential extrapolation to other drug-drug/drug-ethanol interactions. **Conclusions:** Direct interactive effects were not able to be confirmed through this bioinformatics study due to the lack of existing ethanol-acetaminophen simultaneous exposure data. This work suggests that a battery of software applications should be used to assess interactive effects.

## 1. Introduction

### 1.1. Drug-Related Hepatotoxicity

With growing interest in the use of ‘omics’ data and bioinformatics tools to assist clinicians and physician-scientist in decision making, examples of how bioinformatics can be used to support drug-drug or drug-alcohol interaction research are needed [1,2,3]. This paper explores the well-studied interaction of acetaminophen and alcohol as a proof-of-concept for how bioinformatics tools can be used to leverage publically available data to inform decisions when assessing potential drug-ethanol or drug-drug interactive effects. In this paper, the term drug-alcohol interaction specifically references acetaminophen (APAP) and ethanol, respectively. 

Drug-ethanol interaction can result in hepatotoxicity if not closely monitored. Two primary drug-ethanol interactions exist, which, even at a moderate level of ethanol intake, can result in an adverse outcome: (1) Pharmacokinetic interaction in which ethanol interferes with drug metabolism and (2) pharmacodynamic interactions where ethanol enhances the effect of the medication, such as in the nervous system with sedation medication [4].

A number of factors are involved in the production of hepatotoxicity, and understanding the factors that underlie drug-drug or drug-ethanol interaction have particular clinical relevance. One major theme that plays a role in the determination of how drugs may interact is acute or chronic exposure duration. The body may reasonably manage acute and chronic ingestion of APAP or ethanol as individual substances; however, hepatotoxicity becomes a concern when APAP and ethanol are ingested together, when one or the other is still present within the body, or when consumed in extreme excess.

### 1.2. Acetaminophen and Ethanol Metabolism

APAP hepatotoxicity is the result of N-acetyl-p-benzoquinoneimine (NAPQI) buildup. Under normal conditions, ~95% of the drug is metabolized by sulfate and glucuronide pathways, while the remaining 5% is metabolized by cytochrome P-4502E1 (CYP2E1) [5]. However, when consumed in excess, APAP metabolization via sulfate and glucuronide pathways reaches saturation, and the CYP2E1 pathway becomes responsible for metabolization of all remaining APAP. CYP2E1 activity produces the metabolite NAPQI, a reactive oxygen species that binds readily to macromolecules in the liver, hindering functionality and leading to drug-induced liver injury (DILI). Glutathione (GSH) is a molecule found naturally throughout the body, with high concentrations in the liver. GSH will bind and neutralize NAPQI at therapeutic doses and is sufficient to keep the hepatotoxicity potential under control; however, hyperactivity of CYP2E1 resulting from excessive APAP intake will result in depletion of GSH stores. The depletion of GSH stores allows for NAPQI conjugation with liver macromolecules, causing oxidative stress, which can produce hepatic injury [6,7].

Though APAP is designed to be safe at proper therapeutic doses, it is not always necessary for APAP intake to exceed a therapeutic dose to result in hepatotoxicity. Drug-drug or drug-ethanol interactions through competition for metabolic pathways can turn a therapeutic dose into a hepatotoxic dose. Excessive ethanol consumption is also known to result in liver damage, and to some extent, is due to activation of the same metabolic pathway that causes APAP hepatoxicity: CYP2E1 [5]. Chronic ethanol intake has been shown to enhance APAP toxicity by producing a persistent state of upregulated CYP2E1, depleting GSH stores [8].

Five different bioinformatics tools were used to explore the well-known interactive effects of ethanol-acetaminophen exposure as a proof of concept for assessing unknown drug-drug or drug-ethanol interactions. Publicly available tools PharmGKB, Chemical Annotation Retrieval Toolkit, wikipathways, Transcriptome Analysis Console 4.0 (TAC), and STRING were used to observe the known metabolic paths discussed above. In addition, Qiagen, Ingenuity Pathway Analysis, was used for data similarity comparison amongst all bioinformatics tools. Metabolic pathways for each substance were compared to identify pathway activation indicative of either APAP or ethanol exposure, demonstrating that all tools were capable of identifying the potential origin of adverse effects. This work provides supporting evidence that bioinformatics tools can be used to understand interactive pathways following exposure to ethanol and APAP, with potential extrapolation to other drug-drug/drug-ethanol interactions.

## 2. Materials and Methods

### 2.1. Mechanism of Interaction between Acetaminophen and Ethanol

**Figure 1:**
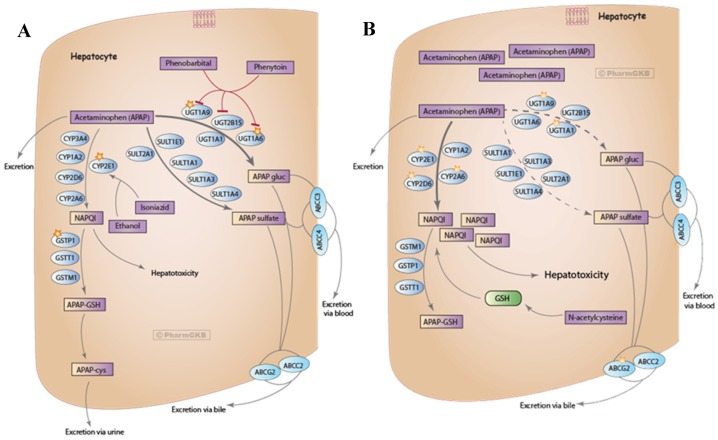
APAP pharmacokinetics for therapeutic doses (**A**) and toxic doses (**B**). The difference between these two figures lies within the relative concentrations of NAPQI, which is a toxic byproduct produced during the metabolism of APAP. The higher the dose of APAP (**B**), the higher the concentration of NAPQI produced in the hepatocyte. The metabolism of APAP to NAPQI is catalyzed by CYP2E1, thus the levels of APAP, NAPQI, and CYP2E1 in the hepatocyte are directly related [19]. This figure has been approved for use under data usage policy from PHARMGKB license policy (https://www.pharmgkb.org/page/dataUsagePolicy).

Acetaminophen (APAP) and ethanol metabolism are well-studied both individually and during combined exposure [9,10,11,12,13,14,15,16]. As individual substances, uninterrupted therapeutic (<4 g/day/adult) doses of APAP follow a well-described path of metabolism through the liver [15]. To summarize, APAP is mostly converted to inactive glucuronide and sulfate conjugates while a fraction is excreted unchanged and the smallest fraction oxidized to NAPQI [15,17]. NAPQI is highly reactive and is the primary contributor of APAP hepatotoxicity [15,17,18]. To avoid hepatotoxicity, NAPQI is typically detoxified through binding to GSH to form APAP-GSH, which is then excreted through urine as shown in Figure 1A [11,15,17,18,19]. However, doses >4 g/day/adult of APAP can result in hepatotoxicity due to saturation of metabolic pathways (glucuronidation), leading to larger amounts of APAP being eliminated unchanged or oxidized into NAPQI [15]. Buildup of NAPQI depletes GSH stores and forms protein adducts that target mitochondrial proteins and ion channels, leading to reduced energy production, ion misbalance, and cell death, as shown in Figure 1B [15,17,18,19,20]. 

**Figure 2:**
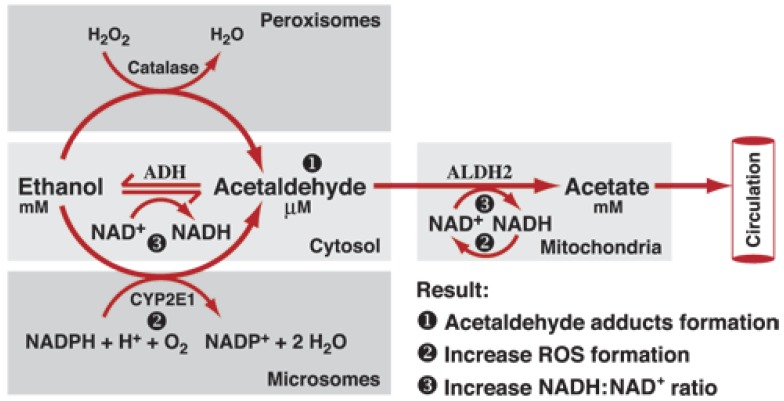
Oxidative pathways of alcohol metabolism. Alcohol dehydrogenase (ADH), cytochrome P450 2E1 (CYP2E1), and catalase all contribute to oxidative metabolism of alcohol. ADH converts alcohol (i.e., ethanol) to acetaldehyde, and at elevated ethanol concentrations, CYP2E1 assists ADH in metabolizing ethanol to acetaldehyde [25]. This figure has been approved for use under copyright information policy from the NIAAA webpage (https://www.niaaa.nih.gov/disclaimer).

Similar to APAP, ethanol has a well-described path of metabolism through oxidation within the liver. The primary enzymes responsible for ethanol oxidation include alcohol dehydrogenase (ADH) and cytochrome P450-dependent ethanol-oxidizing system [21]. ADH is activated at low ethanol concentrations, indicating that ADH activity is sustained until ethanol is eliminated regardless of internal concentration [15]. However, ethanol oxidation is limited by the amount of ADH in the liver which means ADH inhibitors will result in a decrease in ethanol oxidation [15,21]. To assist ADH in ethanol oxidation the Cytochrome P450 2E1 (CYP2E1) enzyme activation is dependent upon higher concentrations of ethanol within the body. Therefore, when ADH is unable to sufficiently oxidize all ethanol present, CYP2E1 activity manages the excess ethanol. Thus, elevated oxidation which exceeds ADH capacity can be attributed to CYP2E1, CYP1A2, and CYP3A4, shown in Figure 2 [15,21]. As APAP metabolism and ethanol metabolism can compete for CYP2E1, combined usage can inhibit metabolism of both substances, prolonging internal concentrations [15,21,22,23,24]. Fortunately, the APAP-ethanol relationship is well documented, so sourcing data to track and identify enzyme activity related to APAP and ethanol metabolism is relatively straightforward. Here, initial steps used publicly available databases and tools to visualize APAP and ethanol metabolism.

### 2.2. Tools to Understand Ethanol-Acetaminophen Interaction

PharmGKB (https://www.pharmgkb.org/), was used as an initial source to identify the known primary pathway for therapeutic and toxic doses of APAP. PHARMGKB is an NIH-funded resource that provides data about how human genetic variation can lead to varied medication responses [15]. PharmGKB is a valuable resource when it comes to understanding potential drug related interactions, as it not only provides informative pharmacokinetic visuals shown in Figure 1A,B, but also gives detailed drug-drug/drug-ethanol interaction information under the prescribing information sections. Additionally, PharmGKB provides clinical guidance on dosing information directly related to genotype [26,27,28]. Genotype-based dosing recommendations are listed in PharmGKB by drug and then by genotype variants. Based on the specific genotype variant, the drug dosing recommendation will deviate from normal recommended values to accommodate an individual with an altered ability to metabolize a specific drug. With a simple search function, PharmGKB identifies shared metabolism mechanisms that could lead to hepatotoxicity, primarily the same CYP450 isoenzymes described earlier (CYP2E1) [15]. With the initial identification and visualization of metabolism completed, secondary analysis was completed confirming shared mechanisms between APAP and ethanol metabolism. Secondary analysis used Galaxy from the Chemical Annotation Retrieval Toolkit (CART) (http://cart.embl.de/).

### 2.3. Application of Bioinformatics Tools

**Figure 3:**
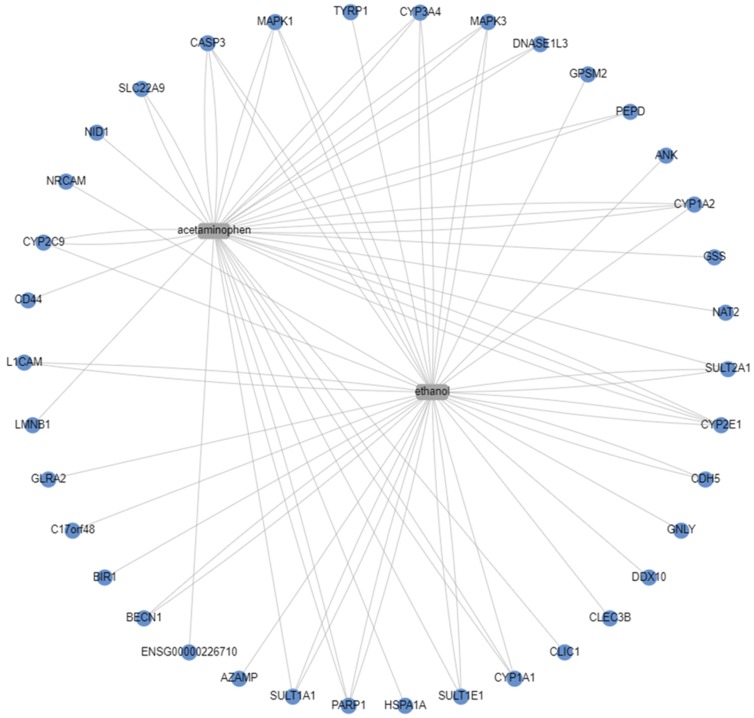
Dynamic network from CART Galaxy visualizing shared genes in the metabolism of ethanol and APAP [29].

CART sources publicly available annotations to find enrichment on the biological effects of chemicals. The CART program is completed through a simple search function by first uploading data. Following data upload, drug annotation databases were selected for analysis. Drug annotation databases searched through CART are funded and curated through various government (USA, Canada, and German), academic (National University of Singapore, NC state University) and non-governmental organizations (World Health Organization) organizations. For this analysis, all available drug annotation databases were selected (drug side effects (SIDER), target proteins (STITCH), Comparative Toxicogenomics Database (CTD), Therapeutic classification levels I, II, and III from the Anatomical Therapeutic Chemical (ATC) classification system, Target proteins (TTD), Therapeutic drug targets (DrugBank), metabolization (DrugBank), Toxicity (DrugMatrix), and functional classification (ChEMBL-FTC)) to provide the broadest range of identifiable interactions, and default settings were used for analysis. Following initial execution of the file, 13 individual analyses were completed. The dynamic network was selected to visualize shared genes between APAP and ethanol metabolism shown in Figure 3. Genes identified in the dynamic network were tabled into three columns using Microsoft Excel: the first column contains genes associated with APAP metabolism, the second column contains genes associated with ethanol metabolism, and the third column contains genes associated with metabolism of both APAP and ethanol. Column 3 was of primary interest, as it provides a succinct list to continue analysis into the metabolic relationship of APAP and ethanol shown in Table 1.

Shared genes between APAP and ethanol identified using CART and PHARMGKB were further analyzed using STRING. STRING is a database comprised of known and predicted protein-protein interactions. Both direct and indirect protein interactions are included in the database. All recorded interactions come from computational prediction, knowledge transfer between organisms, and interactions aggregated from other databases (genomic context predictions, high-throughput lab experiments, conserved co-expression, automated text mining, and previous knowledge in databases) [30,31,32].

For STRING analysis, each gene symbol identified as shared between APAP and ethanol using CART was entered into the STRING website (https://stringdb.org/cgi/input.pl?sessionId=LL53xoM9kuEj&input_page_show_search=on) and *Rattus norvegicus* was selected as the test organism for comparison purposes of later analyses. Initial STRING results displayed limited protein interaction due to the somewhat limited gene input from shared protein analysis in CART. To better understand potential interactions, the “+ more” function was used, which allows additional interactions to be added to the query to better capture complete pathway interactions. Each STRING analysis was then processed using the “clusters” k-means clustering function. In terms of protein-protein interaction, K-means clustering allows for grouping of related proteins by function as long as knowledge of the protein function is already known [33]. Clustering analysis increased visual interpretation of STRING analysis.

### 2.4. Gene Expression Omnibus Data

To move beyond using known interactions as the data input, a raw dataset was sampled from Gene Expression Omnibus (GEO) (GSE122184). GEO is a genomics data repository available for public submission of minimum information about a microarray experiment (MIAME)-compliant data. Multiple analysis tools are imbedded into GEO to assist in the analysis of array and sequence based data. In addition, this data can be exported to external tools for further analysis. To summarize the experimental setup which resulted in the data downloaded from GEO, Male Sprague Dawley rats were exposed to reference hepatotoxicants for five days via oral administration. APAP was one of the hepatotoxicants administered at a concentration of 1000 mg/kg via a carrier of corn oil (2 mL/kg). Additionally, ethanol exposure also occurred at a concentration of 35% ethanol at 2 mL/kg. Following exposure, total RNA was isolated from the left lateral lobe of the liver and analyzed using Affymetrix GeneChip Rat Genome 230_2.0 arrays (https://www.ncbi.nlm.nih.gov/geo/query/acc.cgi?acc=GSE122184). 

For this proof of concept study, only data relating to APAP and ethanol were downloaded and used for comparison. Compressed .CEL microarray files relating to relevant exposures were downloaded from the GEO database and extracted using 7zip. Initial analysis of microarray data used TAC 4.0. TAC 4.0 is software from ThermoFisher scientific designed for biologists to perform detailed analysis of microarray data. TAC allows the user to perform QC, data normalization, statics analysis for differential expression, identify and focus on genes of interest, explore interactions between coding and non-coding RNA and TAC 4.0 provides links to external applications such as wikipathways (https://assets.thermofisher.com/TFS-Assets/LSG/manuals/tac_user_manual.pdf). Files were imported into TAC 4.0 and comparison analyzed using default settings. The comparison analysis compared APAP vs corn oil and ethanol vs corn oil treatments and provided differential expression for rats exposed to either substance. Differential expression between treatments provides insight into the altered transcriptome following exposure to APAP or ethanol. Identified genes that were differentially expressed were then queried for signs of known interaction leading to hepatotoxicity following simultaneous exposure to APAP and ethanol using wikipathways.

Following initial analysis using TAC 4.0, differential expression values from treatment comparisons (ethanol vs corn oil, APAP vs corn oil) were exported into Excel and then imported into IPA for individual treatment analysis. Following data importation, a core analysis was performed for both APAP and ethanol. A comparison analysis between each core analysis was used to identify pathways displaying similar differential expression between APAP and ethanol exposure. Finally, interests were focused on the network analysis that allowed for exploration of interconnected activity across the transcriptome.

## 3. Results

### 3.1. CART Galaxy Analysis

CART Galaxy analysis placed known genes responsible for both APAP and ethanol metabolism into the same figure, shown in Figure 3. This gene diagram was assessed for similarity amongst APAP and ethanol metabolism. A table was created to visualize the shared genes between the two treatments (Table 1).

### 3.2. STRING Analysis

**Figure 4:**
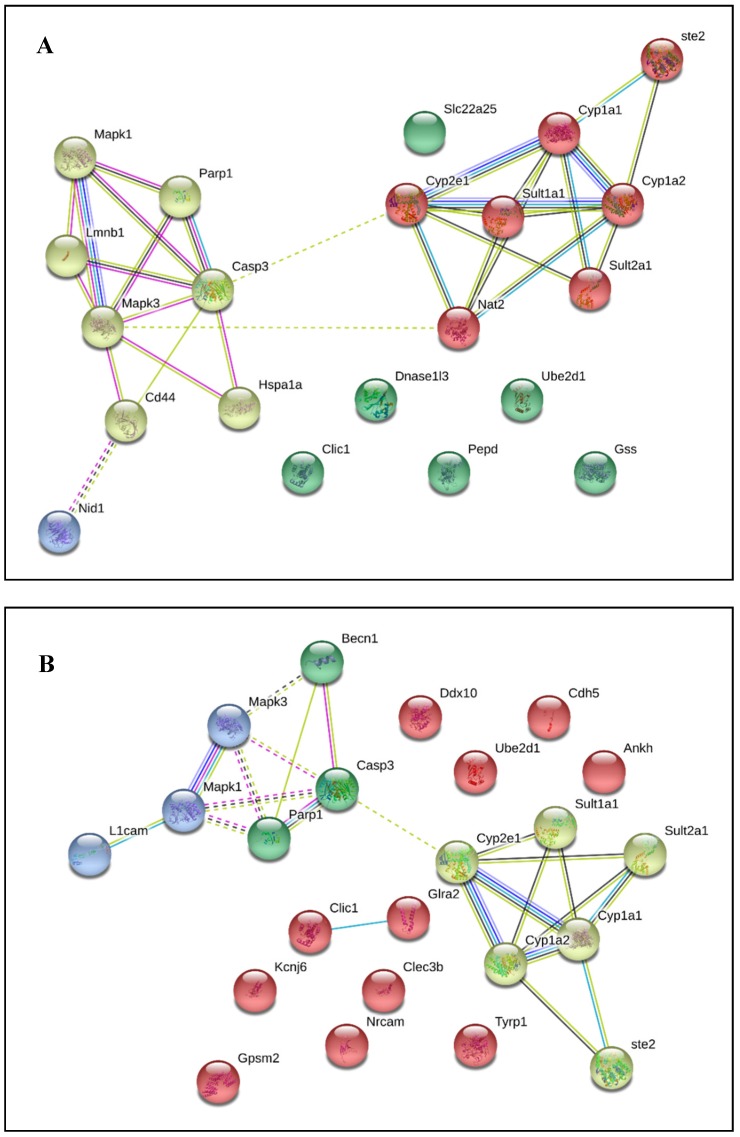
STRING analysis showing genes related to APAP metabolism pathway (**A**) and genes related to ethanol metabolism (**B**). All pathway related genes were identified using CART Galaxy. Colored nodes: query proteins and first shell of interaction. Empty nodes: proteins of unknown 3D structure, filled nodes: 3D structure is known or predicted (This figure is used with permission from Creative Commons Attribution 4.0 License. License agreement: https://creativecommons.org/licenses/by/4.0/legalcode).

STRING analysis was used to visualize the relationship amongst genes related to APAP metabolism and ethanol metabolism individually, shown in Figure 4A,B. The shared genes that were identified in the CART Galaxy analysis that were also visible in the STRING figures were CYP1A1, CYP2E1, SULT1A1, CYP1A2, SULT2A1, CASP3, MAPK3, PARP1, and MAPK1. Additionally, shared genes that were not originally identified as shared between the two metabolism pathways were visible (STE2 and UBE2D1).

### 3.3. Transcriptome Analysis Console 4.0 Dataset Analysis

**Figure 5:**
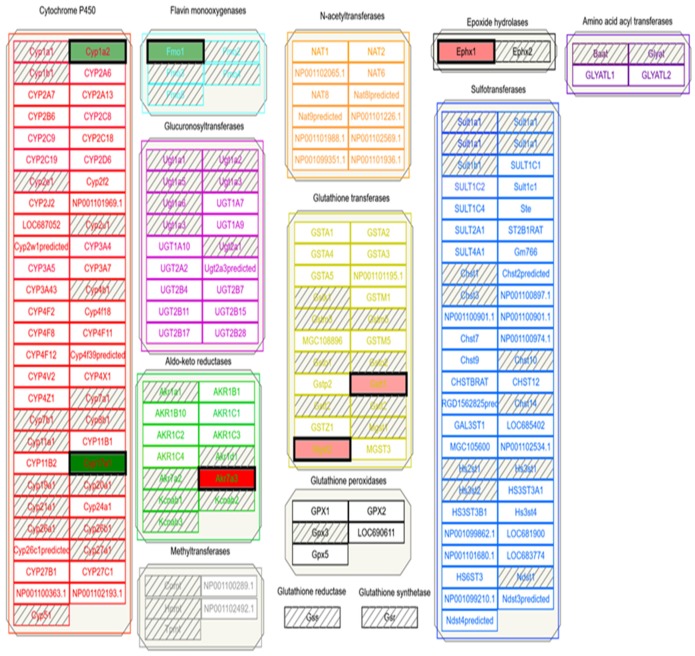
Metabolic pathway of APAP compared to corn oil identified using TAC 4.0 wikipathways link. Individual genes highlighted in green are down-regulated and genes highlighted in red are upregulated. All other genes are present in the metabolic pathway, but were not differentially expressed following the APAP/corn oil comparison. Raw biotransformation file can be found at wikipathways.org by searching for biotransformation (https://www.wikipathways.org/index.php/Pathway:WP1286).

**Figure 6:**
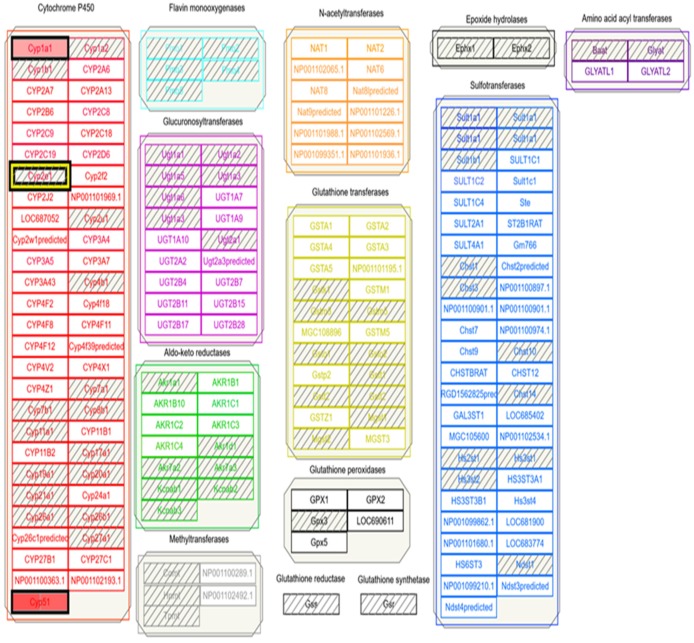
Metabolic pathway of ethanol compared to corn oil identified using TAC 4.0 wikipathways link. Individual genes highlighted in green are down-regulated and green highlighted in red are upregulated. All other genes are present in the metabolic pathway, but were not differentially expressed following the ethanol/corn oil comparison. Raw biotransformation file can be found at wikipathways.org by searching for biotransformation (https://www.wikipathways.org/index.php/Pathway:WP1286).

Following data download from GEO and extraction via 7zip, all data was imported into TAC 4.0 and converted into log 2 fold change expression data. Comparison analysis returned significant differential expression in both up and down-regulated pathways. A portion of differential expression was overlaid into wikipathways to better describe genes involved in APAP and ethanol metabolism from experimental data. The wikipathway, metapathway biotransformation, was selected, and differentially-expressed genes from both APAP and ethanol datasets were compared to the known data described above. Pathway-related genes that were observed in the metapathway for APAP that had also been identified in Figure 1, Figure 3 and Figure 4A,B were CYP1A2, CYP2A6, CYP2D6, CYP3A4, UGT1A9, UGT2B15, GSTM1, GSTT1, and SULT2A1 shown in Figure 5. Pathway related genes that were observed in the metapathway for ethanol that were also identified in Figure 2, Figure 3 and Figure 4 included CYP1A1, CYP2C9, CYP3A4, and SULT2A1, as shown in Figure 6. 

### 3.4. IPA Validation of Pathway Activity

**Figure 7:**
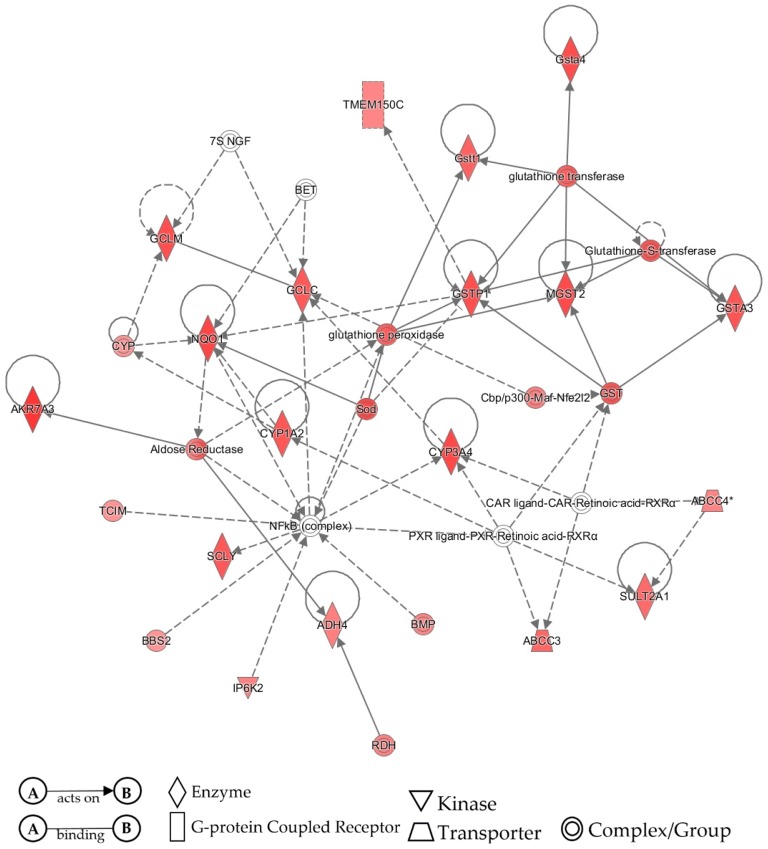
IPA network analysis of APAP metabolism pathway identified from TAC 4.0 dataset of rats exposed to APAP. Nodes shapes identified in the network analysis are vertical diamond (Enzyme), rectangle (G-protein coupled receptor), inverted triangle (kinase), trapezium (transporter), and double circle (complex/group). Solid lines indicate direct interaction and dotted lines indicate indirect association.

**Figure 8:**
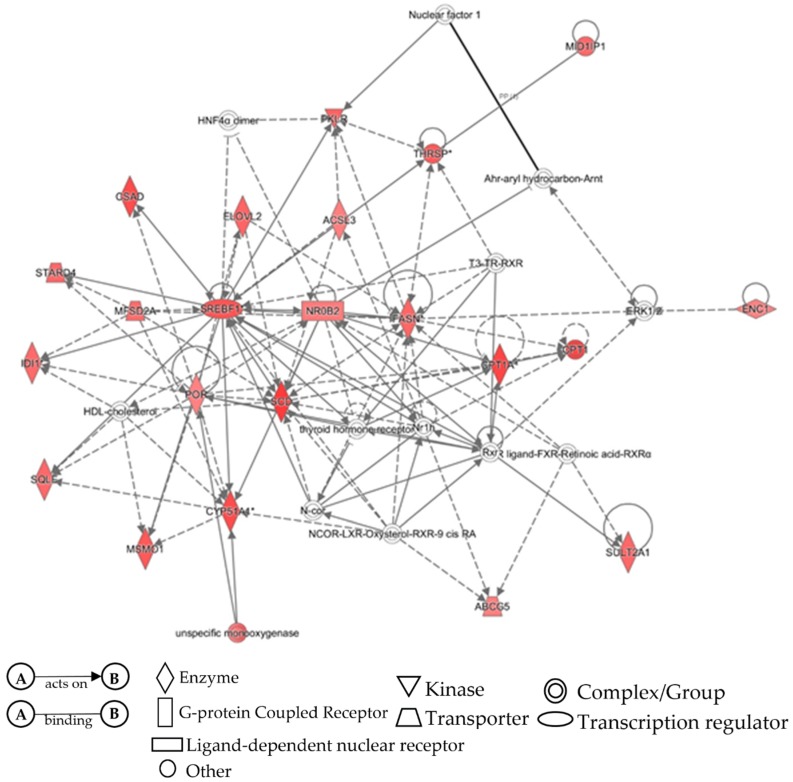
IPA network analysis of ethanol metabolism pathway identified from TAC 4.0 dataset of rats exposed to APAP. Nodes shapes identified in the network analysis are vertical diamond (Enzyme), vertical rectangle (G-protein coupled receptor), horizontal rectangle (ligand-dependent nuclear receptor, oval (transcription regulator), inverted triangle (kinase), trapezium (transporter), single circle (other), and double circle (complex/group). Solid lines indicate direct interaction and dotted lines indicate indirect association.

All differential expression data from the TAC 4.0 analysis was transferred into IPA for network comparison analysis. Both APAP and ethanol were analyzed using separate core analyses. Following the core analysis, a comparison analysis was performed by comparing the differential expression of both APAP and ethanol. Post analysis interest was focused on the network analysis because this would be most similar to the previous information gathered in Figure 1, Figure 2, Figure 3, Figure 4, Figure 5 and Figure 6 and Table 1. From the overall network analysis, the individual nodes most related to metabolic pathways were selected. These are represented by Figure 7 for APAP metabolism and Figure 8 for ethanol metabolism. From the network analysis for APAP, six of the genes in the analysis were identified using the bioinformatics tools discussed earlier (CYP1A2, CYP3A4, SULT2A1, GSTA3, GSTT1, GSTA4) while the ethanol metabolic pathway only shared one gene with previously identified information (SULT2A1), as shown in Figure 8.

## 4. Discussion

The well-documented relationship between APAP and ethanol metabolism was traceable using all bioinformatics tools proposed in this work, with each tool offering a slightly different perspective on the potential degree of interactivity. Initial tools (PHARMGKB, CART, and STRING) leveraged previously known interactive data from NGO, government, and academic databases, so their ability to identify interactions was expected. Tools that leverage publically available databases that are curated by reliable sources are an integral first step when searching for existing data and to compare novel datasets against. Though new drug-drug or drug-alcohol interaction data are unlikely to be found in existing datasets they are valuable tools that may provide easy insight into future work. The PHARMGKB, CART, and STRING tools proved useful as a mirroring step for data generated from the GEO dataset using TAC 4.0 and IPA. All data relating to APAP or ethanol metabolism generated using TAC 4.0 and IPA was validated using PHARMGKB, CART, and STRING by matching genes known to be involved in the metabolic pathways. Having an established knowledge base of information gathered from PHARMGKB, CART, and STRING increased the confidence in data generation using the GEO dataset in TAC 4.0 and IPA. 

Overall, the information generated using each tool was beneficial and could assist during initial drug-drug or drug-ethanol interactive research. CART Galaxy provided the key initial step of identifying genes that were involved in both APAP and ethanol metabolism (CYP3A4, MAPK3, CYP1A2, SULT2A1, CYP2E1, CLIC1, CYP1A1, SULT1A1, CYP2C9, CASP3, and MAPK1). The simple interface allowed direct drug input and selection of the visualization tool to easily identify the genes involved in metabolism shown in Figure 3. Though CART Galaxy provided initial gene interaction data, the analysis may not have captured unseen or less common interactions. Additional gene interactions were explored using STRING. Since the input into STRING was directly related to the output from CART Galaxy, the results were expected to align relatively well. However, STRING offers one function that was not available with CART Galaxy: the “+ more” function. This function enabled the addition of related genes and predicted pathways to further explore potential genes that may be an interaction site following simultaneous exposure to APAP and ethanol. The shared genes that were identified in the CART Galaxy analysis that were also visible in the STRING figures were CYP1A1, CYP2E1, SULT1A1, CYP1A2, SULT2A1, CASP3, MAPK3, PARP1, and MAPK1. Additionally, shared genes that were not originally identified as shared between the two metabolism pathways were visible (STE2 and UBE2D1). 

Following initial exploration of APAP and ethanol metabolism, a dataset from GEO was used to see if genes identified in CART and STRING were also identifiable via gene expression data from rats exposed to either APAP or ethanol. Admittedly, the dataset from GEO was not ideal because the experimental dataset did not include data from rats that were simultaneously exposed to APAP and ethanol. Therefore, the key signature of adverse simultaneous exposure, CYP2E1 activity, was not present and no competition for gene function could be identified. However, the GEO dataset did allow for initial identification of gene activity which may indicate adverse gene expression leading to hepatotoxicity by including datasets from rats exposed to APAP or ethanol individually. TAC 4.0 is specifically designed to analyze Affymetrix arrays. The research team that uploaded the APAP and ethanol rat exposure data utilized Affymetrix assays; therefore, TAC 4.0 was the most logical place to start the raw dataset analysis. 

TAC 4.0 provided valuable insight into overall differential expression when analyzing APAP or ethanol exposure. TAC 4.0 has multiple built-in functionalities accessible by tabs. Within TAC 4.0 tabs is the wikipathways tab which allow the user to specifically select a pathway of interest such as the metapathway biotransformation that was selected for analysis in this research as shown in Ref. Figure 5 and Figure 6. Within the selected pathway, any genes that were differentially expressed in the exposure were highlighted within the wikipathway. Following uploading of the data into TAC4.0, the wikipathway “metapathway biotransformation” was selected for both APAP and ethanol metabolism and genes identified as differentially expressed aligned well with those genes identified in CART and STRING analyses. Again, the genes identified as differentially regulated during the wikipathway analysis were expected. Genes associated with APAP or ethanol metabolism would be expected to be differentially regulated following exposure to APAP or ethanol and therefore captured in the GEO and TAC 4.0 analysis. 

The last remaining step was to use IPA as final validation to see how well all bioinformatics results from varying tools agreed with one another. Differential expression data was exported from TAC 4.0 and imported into IPA. Initial core analyses were performed for APAP and ethanol datasets to set up a comparison analysis. Following the comparison analysis, the network selections most appropriate to metabolic pathways were selected and searched for the same genes identified via CART Galaxy, STRING, and TAC 4.0. A greater number of genes related to APAP metabolism were identified in the IPA network analysis than the network analysis for ethanol metabolism, but overall, both network analyses provided similar insights into genes related to APAP or ethanol metabolism. 

The lack of strong support for identifiable genes in the ethanol exposure is no fault to the bioinformatics software, but to the overall limitations of ‘omics’ based studies. Unless they are rather expansive, ‘omics’ studies can lack the depth of data necessary to fully explore gene activity following exposure to drugs or ethanol. The GEO dataset captured a brief snapshot of how the metabolism of rats respond following exposure to APAP or ethanol by sacrificing the rats at one time-point following exposure. Therefore, the entire metabolic profile cannot be expected to be fully represented, as metabolism is a process that takes time. Rat tissue sampling is limited to a single time-point, and it is not possible to capture the full metabolic profile within one sampling period. Using extrapolation bioinformatics tools such as STRING or IPA, a relatively small dataset can leverage publicly available data to fill gaps by supplementing small datasets with publically available knowledge. Supplementing small datasets with additional genes and pathways provides additional insight into potential sites of interaction that would not be noticed if analysis simply focused the core data extracted from tissue analyses. 

Though IPA and TAC are useful for understanding differential expression, the tools alone do not contain enough information to definitively describe novel drug-drug or drug-ethanol interaction relationships. However, they can be used to explore potential interaction, and with sufficient data from original research, they can be used to identify and to verify drug-drug or drug-ethanol interaction. 

No single bioinformatics tool should be relied upon as a sole source for decision-making, but instead multiple lines of evidence through various bioinformatics tools should be relied upon when trying to understand the complex interactions which occur during simultaneous exposure to various exogenous materials. Though each tool has the ability to confirm already established relationships, such as the case for APAP-ethanol due to the well-established pathways published in the literature, trying to understand novel interactions or related effects would be much more difficult. Bioinformatics tools are only as good as the data available, and if no known interactive data exists to compare results against, it would be difficult to confirm or deny interaction without strong supporting data, which could take years to compile. 

Based on the GEO dataset used as an example to explore APAP-ethanol interaction, the adverse interaction point (CYP2E1) leading to hepatotoxicity would have been missed. The dataset only consisted of rats exposed to either APAP or ethanol individually, but never together. As expected, the measured differential gene expression to either APAP or ethanol exposure was a result of responding to each substance by itself, despite the fact that the adverse interactive response is known to occur following exposure to both substances. In order to assess the interactive identification capability, a public dataset containing exposure to ethanol, APAP, and both ethanol and APAP simultaneously would need to be readily available. However, basic metabolic function related to individual substance exposure is observed throughout the TAC 4.0 analysis as well as in IPA analysis.

Overall, APAP metabolism tracks reasonably well amongst all bioinformatics tools used and ethanol metabolism is detectable across the multiple bioinformatics platforms as well. However, the evidence supporting ethanol metabolism is weaker since a direct tie from experimental data to existing data was not as well supported, but the lack of support is attributed to the experimental design and not necessarily to a fault in the bioinformatics tools found in Figure 2. However, IPA and TAC 4.0 APAP and ethanol metabolism profile results do relate to the CART Galaxy results in Figure 3, providing additional lines of evidence that more than one bioinformatics tool should be used when exploring interactive potential.

## 5. Conclusions

All six bioinformatics tools used in this study capture the metabolism of both APAP and alcohol to some degree. The tools which focused on pre-established data (CART, PHARMGKB, and STRING) were all capable of identifying sites of metabolic interaction which could result in hepatotoxicity. The tools that focused on experimental data (TAC 4.0, wikipathway, and IPA) identified metabolic pathways which were isolated to each individual substance and overall lacked insight into the known sites of interaction (CYP2E1) leading to hepatotoxicity. Ultimately, the lack of insight into metabolic interaction between APAP and alcohol was a function of the experimental data and not the bioinformatics tools. All tools used in this study provided results that indicate they are capable of identifying potential sites of metabolic interaction that could result in an adverse outcome. All bioinformatics tools described in this paper are not limited to existing datasets, but can readily be used to search and describe novel interactions. All tools leverage publically available data so that analysis of novel datasets can be compared to existing datasets with ease. To begin exploration of novel data both IPA and TAC 4.0 are viable options for analysis of omics based data. IPA offers application to the broader omics environment where TAC 4.0 is primarily focused on whole transcriptome microarray. However, novel drug-drug or drug-alcohol experimental assays would need to be developed with corresponding omics samples prior to application of these tools. Any research aiming to apply these tools should begin with predictive interaction tools such as CART and STRING to improve insight and assist in experimental development. Following experimentation the impact of simultaneous exposure can be explored using TAC 4.0 and/or IPA.

## Figures and Tables

**Table 1:** Genes involved in APAP, ethanol, or APAP/ethanol metabolism.

APAP	Ethanol	APAP/Ethanol
	TYRP1	
CYP3A4	CYP3A4	CYP3A4
MAPK3	MAPK3	MAPK3
DNASE1L3		
	GPSM2	
PEPD		
	ANK	
CYP1A2	CYP1A2	CYP1A2
GSS		
NAT2		
SULT2A1	SULT2A1	SULT2A1
CYP2E1	CYP2E1	CYP2E1
	CDH5	
	GNLY	
	DDX10	
	CLEC3B	
CLIC1	CLIC1	CLIC1
CYP1A1	CYP1A1	CYP1A1
SULT1E1	SULT1E1	SULT1E1
HSPA1A		
PARP1	PARP1	PARP1
SULT1A1	SULT1A1	SULT1A1
	AZAMP	
	BECN1	
	BIR1	
	C17ORF48	
	GLRA2	
LMNB1		
	L1CAM	
CD44		
CYP2C9	CYP2C9	CYP2C9
	NRCAM	
NID1		
SLC22A9		
CASP3	CASP3	CASP3
MAPK1	MAPK1	MAPK1
